# Laparoscopic Management of Synchronous Bilateral Ovarian Torsion in a Neonate

**Published:** 2016-01-01

**Authors:** Murat Alkan, Ali Elbek, Cuneyt Evruke, Ahmet Eray Memec, Bulent Aziz Ozkan, Hatice Gülin Sucak, Ozgur Talat Erkan

**Affiliations:** 1 Department of Pediatric Surgery, Division of Pediatric Urology, Cukurova University, Faculty of Medicine, Adana, Turkey; 2 Department of Pediatric Surgery, Medline Adana Hospital, Adana, Turkey; 3 Department of Obstetrics and Gynecology, Cukurova University, Faculty of Medicine, Adana, Turkey; 4 Department of Obstetrics and Gynecology, Medline Adana Hospital, Adana, Turkey; 5 Department of Pediatrics and Neonatology, Medline Adana Hospital, Adana, Turkey; 6 Department of Radiology, Medline Adana Hospital, Adana, Turkey; 7 Department of Anesthesiology, Medline Adana Hospital, Adana, Turkey

**Keywords:** Synchronous, Bilateral ovarian torsion, Neonate

## Abstract

Synchronous bilateral ovarian torsion is an uncommon entity of which both ovaries twist at the same time or observed twisted during the surgical intervention. Herein, we present a neonate with bilateral ovarian torsion, which successfully managed by laparoscopic approach.

## CASE REPORT

A neonate born at term to a 26-year-old primigravida by elective cesarean delivery was diagnosed with an intra-abdominal cystic mass at 35th week of pregnancy. A conservative management plan was undertaken with repeated ultrasonographic evaluations every two weeks. The pregnancy, medical history of the patient and birth were unremarkable. A female full-term infant of 39+5 week of gestation was delivered with a birth weight of 4160 gram. Physical examination was unremarkable. Post-delivery ultrasonography revealed an anechoic uncomplicated cystic mass measuring 5x3.5 cm located in the right pelvis and another semi-solid complicated cystic mass was located in the left pelvis with a measure of 5.4x3.8 cm, without any vascular signal. Both ovaries were not identified. 

Laparoscopy revealed brown round smooth cystic mass with a tiny stalk nearly amputated from the left fallopian tube in the left pelvis. A loop of intestinal segment also twisted around this calcified tiny stalk of the mass (Fig. 1). Right ovary was also twisted with the cystic mass (Fig. 2). The right ovary was untwisted 2 times (360 degree) and left in the pelvis after cyst aspiration without excision and ovarian fixation. Then the left ovary examined and seemed to be nearly amputated from the adnexa. It was removed after cyst aspiration. The calcified stalk was separated from the bowel. The post-operative period was uneventful and the patient was discharged on the 2nd post-operative day. Follow-up at 1 month with ultrasonographic evaluations showed an18 mm right ovary and no cyst.

**Figure:**
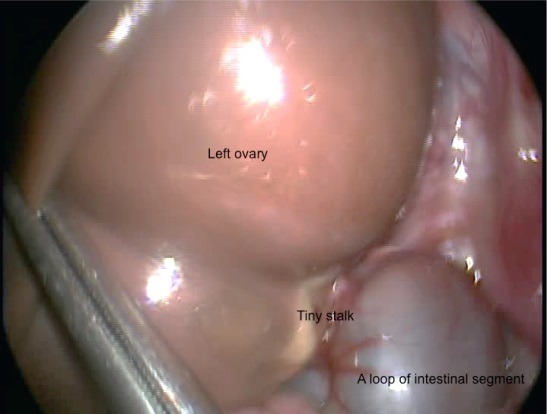
Figure 1: A loop of intestinal segment twisted around the calcified stalk of the left brownish ovarian cyst.

**Figure:**
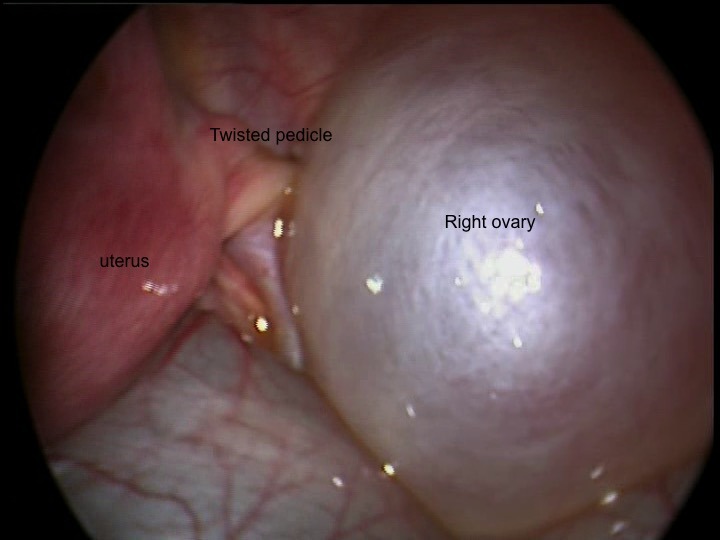
Figure 2: Twisted right ovarian cyst.

## DISCUSSION

The differential diagnosis of a prenatal cystic abdominal mass includes omental cyst, mesenteric cyst, intestinal duplication, urachal cysts, renal cysts and ovarian cysts [1]. Preoperative diagnosis of ovarian torsion is difficult not only in newborns or infants but also in pre pubertal girls. The etiology of the ovarian cysts during pregnancy is related to the immature hypothalamus-pituitary-ovarian feedback that results gonadal hyperstimulation or placental insufficiency with incomplete maturation of the gonadostat. There are few reported patients of asynchronous bilateral adnexal torsion in children in the literature [2, 3]. Synchronous bilateral ovarian torsion has also been reported which was successfully managed by laparoscopic detorsion [4]. 

When a post-natal radiological evaluation reveals a complicated ovarian cyst, irrespective of the size, surgical management recommended either by laparoscopy or laparotomy [5]. Uncomplicated cysts, smaller than 5 cm diameter usually resolve spontaneously while the cysts exceeding 5 cm should be decompress via aspiration to prevent complications [6]. This case highlights the importance of high index of suspicion and early diagnosis of bilateral ovarian torsion in the newborn with bilateral pelvic cysts detected on the pre-natal and post-natal scans.

## Footnotes

**Source of Support:** Nil

**Conflict of Interest:** Nil
